# China enters the global vaccine market 

**DOI:** 10.2471/BLT.14.020914

**Published:** 2014-09-01

## Abstract

China is gearing up to supply the world with affordable vaccines that fulfil all efficacy, safety and quality requirements. Jane Parry reports.

The global vaccine industry has long been dominated by a few multinational companies. But now that companies in China, India and other emerging economies are becoming major vaccine manufacturers and have started selling these vaccines on the international market, competition is set to increase and prices to come down.

For Jiankang Zhang, representative for PATH’s China Programmes, the growth of China’s vaccine industry is “a very positive development for global health, as governments and international procurement agencies will be able to afford more life-saving vaccines and thus protect more lives”.

Since 1987, vaccine quality for international procurement has been assured through the prequalification system that is managed by the World Health Organization (WHO). The “prequalified” stamp of approval means that these vaccines are consistently safe, effective and of high quality, and thus recommended for bulk purchase by the United Nations Children’s Fund (UNICEF) in 152 low and middle-income countries, the GAVI Alliance – which funds vaccines in 73 of these countries – and other agencies.

**Figure:**
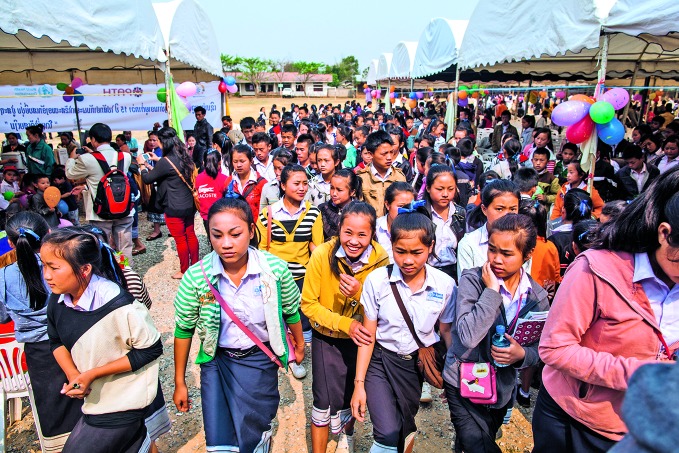
Children gather to be vaccinated for Japanese encephalitis in the Xiangkhouang province of Lao People's Democratic Republic

PATH/Aaron Joel Santos

When WHO pre-qualified a Chinese-made vaccine for the first time last October, the move showed what Chinese vaccine manufacturers could potentially achieve and – in a sense – paved the way for others to follow suit. 

“The prequalification of the Japanese encephalitis vaccine in China is a big step forward, and now several other Chinese producers are interested in obtaining prequalification for their vaccines,” says Melissa Malhame, whose team at the GAVI Secretariat in Geneva works with vaccine manufacturers around the world to ensure sufficient supply of high quality vaccines at affordable prices.

This and other Chinese vaccines are licensed by the China Food and Drug Administration (CFDA) that is part of China’s National Regulatory Authority (CNRA), which received WHO’s seal of approval in March 2011, after finding that it met WHO standards for vaccine regulatory oversight.

In July, this status was renewed, after a successful WHO reassessment of the vaccine regulatory part of the CNRA. WHO Director-General Dr Margaret Chan welcomed the news saying: “As a result of this evaluation, WHO is confident in the quality, safety and effectiveness of vaccines that are made in China.”

For Dr Lance Rodewald, head of WHO’s expanded programme on immunization (EPI) in China, the two WHO programmes – prequalification and national regulatory authority strengthening – “are really terrific, as they have made it possible for the United Nations and other agencies to procure life-saving vaccines for countries without the capacity to make high quality vaccines or the resources to purchase them”. 

“More Chinese vaccine manufacturers will follow suit and apply for pre-qualification later this year or early next year,” Rodewald says.

“As a result of this evaluation, WHO is confident in the quality, safety and effectiveness of vaccines that are made in China.”

Margaret Chan

China has come a long way since 1978 when it introduced EPI and a largely state-run vaccine manufacturing industry grew up to meet the resulting demand.

Initially, the programme offered the country’s children vaccines for polio, diphtheria, tetanus, pertussis (whooping cough), measles and tuberculosis. Later vaccines for hepatitis A and B, Japanese encephalitis, mumps, rubella and invasive meningococcal disease were added.

According to the CFDA, China has 34 vaccine manufacturers, of which four are international joint ventures, seven are state run and the rest are private. All 34, it says, have met the most recent (2010) Good Manufacturing Practices requirements. 

“China is currently producing nearly all of the commonly-used vaccines for viral diseases such as influenza, measles, rabies (for humans), mumps, rotavirus, hepatitis A and B and for bacterial diseases, including typhoid, tetanus and diphtheria,” says Dr Xu Ming, Vice President of the China Chamber of Commerce for Import and Export of Medicines and Health Products.

The dominant player in China’s vaccine industry is state-owned Sinopharm’s subsidiary China National Biotechnology Group, comprising seven manufacturers – one of which is the Chengdu Institute of Biological Products, the producer of the Japanese encephalitis vaccine that was prequalified last year.

In 2010, the China National Biotechnology Group supplied over 740 million doses of 34 vaccines for 28 diseases, representing more than 85% of the vaccines used in China’s immunization programme. The remaining vaccines are purchased by middle-class Chinese families. 

China has been exporting vaccines for diphtheria, hepatitis B, measles, pertussis, polio, tetanus and yellow fever to poor countries for decades through bilateral aid programmes, says Xu, but “WHO prequalification of the Japanese encephalitis vaccine is a game changer.”

“As more Chinese companies obtain WHO prequalification, this will open the door to the export of Chinese vaccines,” Xu says.

“As more Chinese companies obtain WHO prequalification, this will open the door to the export of Chinese vaccines.”

Xu Ming

The CFDA’s role is vital to this process, as it looks at whether the clinical trials needed for licensing a vaccine are conducted ethically and meet international standards. The CFDA only licenses vaccine once it is satisfied with all the required data, including from clinical trials and technical reviews of production facilities and processes, provided by the manufacturer. 

Once a new vaccine is licensed, every lot is chemically and biologically tested before it is released along the supply chain – a lot-release system that has been in place since the mid-2000s.

Having a national regulatory agency that meets international standards lets the purchasers and users of the vaccine know that the quality, safety and effectiveness of the vaccine are assured, Rodewald explains.

A major challenge for China’s vaccine industry is to overcome concerns about product safety. China has been plagued in recent years by scandals over drug and food safety. In 2007, the former head of the Chinese State Food and Drug Administration was sentenced to death and executed, after being found guilty of taking bribes and failing to ensure the safety of drugs and devices approved during his tenure.

At home, the Chinese vaccine industry must overcome its image problems, especially with middle-class parents who can afford to pay out-of-pocket for vaccinations and, therefore, exercise a choice about where they are from.

This is important – not just for the sake of confidence at home – but because domestic use will generate vital post-marketing data for vaccine-related adverse events, as these data are needed in countries ill equipped to do such surveillance. There are always some adverse events and the knowledge gained from these enables manufacturers to improve their products. 

For Xu, several things are needed to restore public confidence. These include improved information sharing and communication between authorities to identify problems or risks, adjust and upgrade standards and improve the quality of vaccines. The industry also needs to step up its post-marketing surveillance while more stringent regulation is needed.

Betty Su, vice president for Asia Pacific, Boston Health care Associates, Inc., a firm specialized in global market access issues for the health-care industry, echoes Xu’s point about post-marketing surveillance.

“The Chinese industry must realize that quality isn't just about releasing one batch of safe products but about safe transportation – something that is also important when China starts to supply the world with vaccines. This entails, among other things, establishing a tracking and monitoring system of the product's quality when it is out there being given to patients.”

But what would it take for more Chinese vaccines to be marketed outside China? Prequalification is one factor, but can be challenging for manufacturers. The Chengdu Institute of Biological Products obtained WHO prequalification for its Japanese encephalitis vaccine after years of wide-ranging technical support from nongovernmental agency PATH, which is funded by the Bill & Melinda Gates Foundation.

The Clinton Health Access Initiative (CHAI) has been working with Chinese suppliers to support their applications for WHO prequalification for several vaccine candidates for the last two years, says Joshua Chu, CHAI’s Director, Vaccines Markets.

The initiative also works with donors and other agencies to implement innovative market-based mechanisms, such as long-term volume guarantees, to make donor funds go further when purchasing vaccines. So far it has established volume guarantees with vaccine manufacturers in India and with other developing countries for other health products. 

After Japanese encephalitis, there are plenty of good potential candidates for prequalification and purchase by United Nations agencies, says GAVI’s Malhame. “These include pneumococcal conjugate, rotavirus, human papillomavirus, meningitis A, measles-rubella and inactivated polio virus (IPV) vaccines. China’s vaccine industry should also be encouraged to enter international markets with WHO-prequalified products for other vaccines that are not supported by GAVI.”

**Figure:**
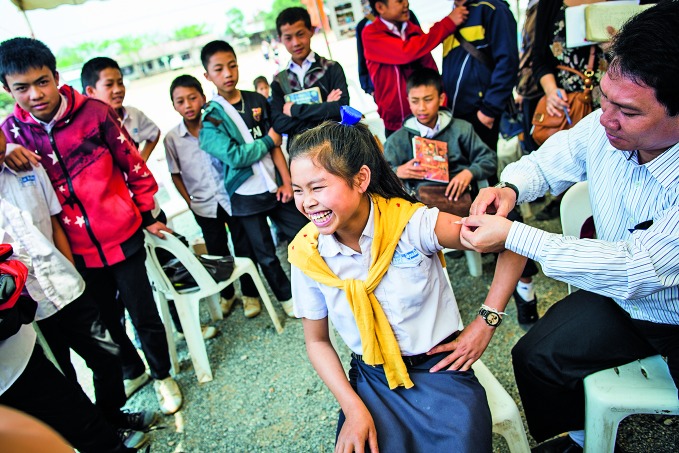
Japanese encephalitis vaccination campaign in the Xiangkhouang province of Lao People's Democratic Republic

PATH/Aaron Joel Santos

Rodewald is also keen to see China’s new IPV vaccine coming down the prequalification pipeline, since the Global Polio Eradication Initiative’s Eradication and Endgame Strategic Plan 2013–18 calls for the vaccine to be introduced into immunization programmes by the end of 2015.

Last year GAVI said it would support the introduction of IPV in the 73 countries that are eligible for its support at about US$ 1 a dose in 10-dose vials. For middle-income countries, UNICEF recently said it would offer the vaccine at between US$ 2 and US$ 3.30 per dose for the same 10-dose package.

“Twenty years ago China began a Sabin strain IPV development programme, and this is likely to be licensed by the CFDA this year,” says Rodewald. “This will be only the second Sabin IPV to be licensed worldwide and would be a very good candidate for WHO prequalification.”

